# Supplementary material for: Radiocaesium accumulation capacity of epiphytic lichens and adjacent barks collected at the perimeter boundary site of the Fukushima Dai-ichi Nuclear Power Station
Source: PLoS One. 2021 May 24;16(5):e0251828. doi: 10.1371/journal.pone.0251828 (PMC8143426; doi:10.1371/journal.pone.0251828)
Supplement: S1 Table — Each data was decay corrected to the sampling date. The uncertainty values were included both counting errors and efficiency calibration errors. The 134Cs/137Cs ratios were obtained by decay-correcting the date to 11 March 2011. (PDF) [file pone.0251828.s001.pdf]

S1 Table. Radiocaesium activity concentrations and inventories of all samples by gamma spectroscopy.

Substrate (tree)	Sample type	Sample ID	Species (for lichens)	Sampling date	Radiocaesium activity concentrations (kBq kg^{-1})		Radiocaesium inventories (kBq m^{-2})		Activity ratio ^{134}Cs/^{137}Cs
					^{134}Cs	^{137}Cs	^{134}Cs	^{137}Cs	
Zelkova 1	lichen	Z1-L1	PuB	2017/6/22	4.61E+02 ± 6.94E+00	3.07E+03 ± 2.27E+01	1.12E+02 ± 1.69E+00	7.48E+02 ± 5.53E+00	1.1
Zelkova 1	lichen	Z1-L2	PC	2017/6/22	2.01E+02 ± 3.72E+00	1.33E+03 ± 1.10E+01	4.61E+01 ± 8.53E-01	3.05E+02 ± 2.51E+00	1.1
Zelkova 1	lichen	Z1-L3	PuB	2017/6/22	3.61E+02 ± 5.41E+00	2.41E+03 ± 1.78E+01	9.00E+01 ± 1.35E+00	6.01E+02 ± 4.44E+00	1.1
Zelkova 1	lichen	Z1-L4	PuB	2017/6/22	2.23E+02 ± 4.47E+00	1.56E+03 ± 1.33E+01	4.87E+01 ± 9.78E-01	3.41E+02 ± 2.91E+00	1.0
Zelkova 1	lichen	Z1-L5	PC	2017/6/22	4.11E+02 ± 6.24E+00	2.77E+03 ± 2.06E+01	9.57E+01 ± 1.45E+00	6.45E+02 ± 4.79E+00	1.1
Zelkova 1	lichen	Z1-L6	PuB	2017/6/22	4.25E+02 ± 6.61E+00	2.92E+03 ± 2.18E+01	8.67E+01 ± 1.35E+00	5.96E+02 ± 4.44E+00	1.0
Zelkova 1	lichen	Z1-L7	DA	2017/6/22	3.04E+02 ± 6.36E+00	2.03E+03 ± 1.80E+01	5.14E+01 ± 1.08E+00	3.44E+02 ± 3.05E+00	1.1
Zelkova 1	lichen	Z1-L8	PuB	2017/6/22	3.94E+02 ± 5.71E+00	2.69E+03 ± 1.94E+01	1.08E+02 ± 1.57E+00	7.41E+02 ± 5.35E+00	1.0
Zelkova 1	lichen	Z1-L9	PuB	2017/6/22	3.63E+02 ± 7.01E+00	2.51E+03 ± 2.10E+01	6.51E+01 ± 1.26E+00	4.50E+02 ± 3.76E+00	1.0
Zelkova 1	lichen	Z1-L10	PuB	2017/6/22	4.04E+02 ± 7.08E+00	2.76E+03 ± 2.18E+01	8.02E+01 ± 1.41E+00	5.48E+02 ± 4.33E+00	1.0
Zelkova 1	lichen	Z1-L11	PC	2017/6/22	5.11E+02 ± 8.89E+00	3.36E+03 ± 2.69E+01	7.53E+01 ± 1.31E+00	4.95E+02 ± 3.96E+00	1.1
Zelkova 1	lichen	Z1-L12	DA	2017/6/22	6.39E+02 ± 1.09E+01	4.38E+03 ± 3.41E+01	1.04E+02 ± 1.78E+00	7.12E+02 ± 5.55E+00	1.0
Zelkova 1	lichen	Z1-L13	PC	2017/6/22	4.49E+02 ± 7.47E+00	3.02E+03 ± 2.33E+01	6.91E+01 ± 1.15E+00	4.64E+02 ± 3.59E+00	1.1
Zelkova 1	lichen	Z1-L14	PC	2017/6/22	2.24E+02 ± 5.86E+00	1.63E+03 ± 1.61E+01	3.26E+01 ± 8.53E-01	2.37E+02 ± 2.35E+00	0.98
Zelkova 1	lichen	Z1-L15	PuB	2017/6/22	4.11E+02 ± 5.69E+00	2.84E+03 ± 2.02E+01	9.98E+01 ± 1.38E+00	6.90E+02 ± 4.92E+00	1.0
Zelkova 1	lichen	Z1-L16	PC	2017/6/22	3.64E+02 ± 6.82E+00	2.51E+03 ± 2.08E+01	5.87E+01 ± 1.10E+00	4.05E+02 ± 3.35E+00	1.0
Zelkova 1	lichen	Z1-L17	PuB	2017/6/22	2.74E+02 ± 5.90E+00	1.93E+03 ± 1.72E+01	4.67E+01 ± 1.01E+00	3.29E+02 ± 2.94E+00	1.0
Zelkova 2	lichen	Z2-L1	PC	2017/6/23	2.55E+02 ± 4.46E+00	1.75E+03 ± 1.39E+01	5.45E+01 ± 9.53E-01	3.74E+02 ± 2.98E+00	1.0
Zelkova 2	lichen	Z2-L2	PC	2017/6/23	2.09E+02 ± 3.77E+00	1.37E+03 ± 1.12E+01	5.91E+01 ± 1.07E+00	3.88E+02 ± 3.16E+00	1.1
Zelkova 2	lichen	Z2-L3	PA	2017/6/23	1.48E+02 ± 3.96E+00	1.03E+03 ± 1.06E+01	2.87E+01 ± 7.69E-01	2.00E+02 ± 2.05E+00	1.0
Zelkova 2	lichen	Z2-L4	PC	2017/6/23	3.03E+02 ± 5.12E+00	2.09E+03 ± 1.63E+01	7.56E+01 ± 1.28E+00	5.22E+02 ± 4.06E+00	1.0
Zelkova 2	lichen	Z2-L5	PC	2017/6/23	2.34E+02 ± 5.61E+00	1.68E+03 ± 1.58E+01	4.05E+01 ± 9.72E-01	2.91E+02 ± 2.74E+00	1.0
Zelkova 2	lichen	Z2-L6	PM	2017/6/23	3.19E+02 ± 5.49E+00	2.21E+03 ± 1.73E+01	7.64E+01 ± 1.31E+00	5.29E+02 ± 4.15E+00	1.0
Zelkova 2	lichen	Z2-L7	PuB	2017/6/23	1.58E+02 ± 3.36E+00	1.10E+03 ± 9.72E+00	4.67E+01 ± 9.94E-01	3.25E+02 ± 2.87E+00	1.0
Zelkova 2	lichen	Z2-L8	PuB	2017/6/23	1.45E+02 ± 3.46E+00	9.60E+02 ± 9.29E+00	3.73E+01 ± 8.90E-01	2.47E+02 ± 2.39E+00	1.1
Zelkova 2	lichen	Z2-L9	PC	2017/6/23	7.92E+02 ± 1.23E+01	5.47E+03 ± 4.07E+01	1.17E+02 ± 1.82E+00	8.10E+02 ± 6.03E+00	1.0
Zelkova 2	lichen	Z2-L10	PuB	2017/6/23	4.06E+02 ± 7.43E+00	2.80E+03 ± 2.27E+01	6.74E+01 ± 1.23E+00	4.65E+02 ± 3.77E+00	1.0
Zelkova 2	lichen	Z2-L11	PuB	2017/6/23	2.61E+02 ± 4.91E+00	1.76E+03 ± 1.46E+01	5.65E+01 ± 1.06E+00	3.81E+02 ± 3.16E+00	1.1
Zelkova 2	lichen	Z2-L12	PuB	2017/6/23	2.99E+02 ± 5.73E+00	2.06E+03 ± 1.71E+01	5.36E+01 ± 1.03E+00	3.69E+02 ± 3.07E+00	1.0
Zelkova 2	lichen	Z2-L13	CA	2017/6/23	2.53E+02 ± 5.57E+00	1.71E+03 ± 1.55E+01	4.52E+01 ± 9.96E-01	3.06E+02 ± 2.78E+00	1.1
Zelkova 2	lichen	Z2-L14	PuB	2017/6/23	2.43E+02 ± 6.07E+00	1.72E+03 ± 1.67E+01	3.33E+01 ± 8.31E-01	2.36E+02 ± 2.28E+00	1.0
Zelkova 3	lichen	Z3-L1	PuB	2017/6/23	3.58E+02 ± 5.45E+00	2.39E+03 ± 1.79E+01	8.79E+01 ± 1.34E+00	5.87E+02 ± 4.39E+00	1.1
Zelkova 3	lichen	Z3-L2	PuB	2017/6/23	4.08E+02 ± 5.87E+00	2.76E+03 ± 2.01E+01	1.05E+02 ± 1.51E+00	7.10E+02 ± 5.16E+00	1.1
Zelkova 3	lichen	Z3-L3	PC	2017/6/23	3.29E+02 ± 4.51E+00	2.22E+03 ± 1.58E+01	1.07E+02 ± 1.47E+00	7.24E+02 ± 5.16E+00	1.1
Zelkova 3	lichen	Z3-L4	PuB	2017/6/23	2.74E+02 ± 4.55E+00	1.84E+03 ± 1.43E+01	6.63E+01 ± 1.10E+00	4.45E+02 ± 3.47E+00	1.1
Zelkova 3	lichen	Z3-L5	ML	2017/6/23	9.78E+02 ± 1.17E+01	6.70E+03 ± 4.50E+01	1.91E+02 ± 2.29E+00	1.31E+03 ± 8.81E+00	1.0
Zelkova 3	lichen	Z3-L6	PuB	2017/6/23	3.43E+02 ± 6.77E+00	2.35E+03 ± 2.01E+01	5.41E+01 ± 1.07E+00	3.71E+02 ± 3.17E+00	1.0
Zelkova 3	lichen	Z3-L7	DA	2017/6/23	5.35E+02 ± 9.29E+00	3.67E+03 ± 2.91E+01	7.70E+01 ± 1.34E+00	5.28E+02 ± 4.19E+00	1.0
Zelkova 3	lichen	Z3-L8	ML	2017/6/23	3.63E+02 ± 6.36E+00	2.56E+03 ± 2.02E+01	7.27E+01 ± 1.27E+00	5.13E+02 ± 4.05E+00	1.0
Zelkova 1	bark	Z1-B1	-	2017/6/22	8.91E+00 ± 4.20E-01	6.00E+01 ± 9.58E-01	1.01E+01 ± 4.75E-01	6.79E+01 ± 1.08E+00	1.1
Zelkova 1	bark	Z1-B2	-	2017/6/22	1.52E+01 ± 4.21E-01	1.05E+02 ± 1.12E+00	2.53E+01 ± 7.01E-01	1.75E+02 ± 1.86E+00	1.0
Zelkova 1	bark	Z1-B3	-	2017/6/22	2.12E+00 ± 1.37E-01	1.54E+01 ± 3.02E-01	4.75E+00 ± 3.06E-01	3.45E+01 ± 6.76E-01	0.98
Zelkova 1	bark	Z1-B4	-	2017/6/22	7.61E-01 ± 1.32E-01	5.68E+00 ± 2.36E-01	1.28E+00 ± 2.22E-01	9.54E+00 ± 3.97E-01	0.96
Zelkova 1	bark	Z1-B5	-	2017/6/22	8.10E+00 ± 2.67E-01	6.05E+01 ± 7.04E-01	1.66E+01 ± 5.47E-01	1.24E+02 ± 1.44E+00	0.96
Zelkova 1	bark	Z1-B6	-	2017/6/22	5.29E+00 ± 2.11E-01	3.67E+01 ± 5.06E-01	1.20E+01 ± 4.80E-01	8.34E+01 ± 1.15E+00	1.0
Zelkova 1	bark	Z1-B7	-	2017/6/22	6.55E+00 ± 2.93E-01	4.35E+01 ± 6.85E-01	9.75E+00 ± 4.37E-01	6.48E+01 ± 1.02E+00	1.1
Zelkova 1	bark	Z1-B8	-	2017/6/22	7.62E+00 ± 2.70E-01	5.21E+01 ± 6.63E-01	1.73E+01 ± 6.13E-01	1.19E+02 ± 1.51E+00	1.0
Zelkova 1	bark	Z1-B9	-	2017/6/22	7.54E+00 ± 3.19E-01	5.06E+01 ± 7.52E-01	1.49E+01 ± 6.29E-01	9.98E+01 ± 1.48E+00	1.1
Zelkova 1	bark	Z1-B10	-	2017/6/22	2.48E+01 ± 5.40E-01	1.73E+02 ± 1.55E+00	4.38E+01 ± 9.54E-01	3.06E+02 ± 2.74E+00	1.0
Zelkova 1	bark	Z1-B11	-	2017/6/22	4.76E+00 ± 3.36E-01	3.31E+01 ± 7.11E-01	5.56E+00 ± 3.93E-01	3.87E+01 ± 8.31E-01	1.0
Zelkova 2	bark	Z2-B1	-	2017/6/23	1.42E+00 ± 1.75E-01	1.13E+01 ± 3.53E-01	2.31E+00 ± 2.85E-01	1.84E+01 ± 5.74E-01	0.90
Zelkova 2	bark	Z2-B2	-	2017/6/23	1.97E+01 ± 7.11E-01	1.41E+02 ± 1.79E+00	2.14E+01 ± 7.74E-01	1.53E+02 ± 1.95E+00	1.0
Zelkova 2	bark	Z2-B3	-	2017/6/23	7.48E+00 ± 4.14E-01	5.50E+01 ± 9.50E-01	8.92E+00 ± 4.94E-01	6.56E+01 ± 1.13E+00	0.97
Zelkova 2	bark	Z2-B4	-	2017/6/23	3.43E+00 ± 4.02E-01	2.23E+01 ± 7.41E-01	3.49E+00 ± 4.10E-01	2.27E+01 ± 7.55E-01	1.1
Zelkova 2	bark	Z2-B5	-	2017/6/23	1.09E+00 ± 1.51E-01	8.50E+00 ± 2.98E-01	1.78E+00 ± 2.47E-01	1.39E+01 ± 4.86E-01	0.92
Zelkova 2	bark	Z2-B6	-	2017/6/23	9.24E+00 ± 5.05E-01	6.37E+01 ± 1.15E+00	1.17E+01 ± 6.36E-01	8.03E+01 ± 1.45E+00	1.0
Zelkova 2	bark	Z2-B7	-	2017/6/23	7.67E+00 ± 3.79E-01	5.67E+01 ± 9.15E-01	9.89E+00 ± 4.88E-01	7.31E+01 ± 1.18E+00	0.97
Zelkova 2	bark	Z2-B8	-	2017/6/23	8.90E+00 ± 4.70E-01	6.16E+01 ± 1.09E+00	7.53E+00 ± 3.98E-01	5.21E+01 ± 9.21E-01	1.0
Zelkova 2	bark	Z2-B9	-	2017/6/23	1.56E+01 ± 5.89E-01	1.13E+02 ± 1.50E+00	1.82E+01 ± 6.86E-01	1.32E+02 ± 1.75E+00	0.99
Zelkova 2	bark	Z2-B10	-	2017/6/23	2.82E+00 ± 1.97E-01	2.06E+01 ± 4.51E-01	4.51E+00 ± 3.14E-01	3.29E+01 ± 7.20E-01	0.98
Zelkova 2	bark	Z2-B11	-	2017/6/23	5.11E+00 ± 3.37E-01	3.22E+01 ± 7.15E-01	5.90E+00 ± 3.90E-01	3.72E+01 ± 8.26E-01	1.1
Zelkova 3	bark	Z3-B1	-	2017/6/23	6.18E+00 ± 2.45E-01	4.45E+01 ± 6.13E-01	1.30E+01 ± 5.15E-01	9.36E+01 ± 1.29E+00	0.99
Zelkova 3	bark	Z3-B2	-	2017/6/23	5.81E+00 ± 2.72E-01	4.40E+01 ± 6.70E-01	8.92E+00 ± 4.18E-01	6.75E+01 ± 1.03E+00	0.94
Zelkova 3	bark	Z3-B3	-	2017/6/23	3.68E+01 ± 7.29E-01	2.56E+02 ± 2.19E+00	5.45E+01 ± 1.08E+00	3.79E+02 ± 3.24E+00	1.0
Zelkova 3	bark	Z3-B4	-	2017/6/23	2.36E+01 ± 5.88E-01	1.64E+02 ± 1.59E+00	3.78E+01 ± 9.42E-01	2.63E+02 ± 2.55E+00	1.0
Zelkova 3	bark	Z3-B5	-	2017/6/23	1.38E+01 ± 4.79E-01	9.81E+01 ± 1.21E+00	1.89E+01 ± 6.56E-01	1.34E+02 ± 1.65E+00	1.0
Cerasus 1	lichen	C1-L1	PA	2017/7/14	2.59E+02 ± 4.98E+00	1.78E+03 ± 1.49E+01	5.49E+01 ± 1.06E+00	3.77E+02 ± 3.15E+00	1.1
Cerasus 1	lichen	C1-L2	PA	2017/7/14	2.28E+02 ± 5.27E+00	1.58E+03 ± 1.48E+01	3.54E+01 ± 8.19E-01	2.45E+02 ± 2.30E+00	1.1
Cerasus 1	lichen	C1-L3	PM	2017/7/14	2.81E+02 ± 5.22E+00	1.93E+03 ± 1.58E+01	6.74E+01 ± 1.25E+00	4.63E+02 ± 3.80E+00	1.1
Cerasus 1	lichen	C1-L4	PT	2017/7/14	8.08E+01 ± 2.76E+00	5.63E+02 ± 6.95E+00	1.45E+01 ± 4.96E-01	1.01E+02 ± 1.25E+00	1.0
Cerasus 1	lichen	C1-L5	PA	2017/7/14	1.20E+02 ± 5.29E+00	7.86E+02 ± 1.22E+01	1.75E+01 ± 7.71E-01	1.14E+02 ± 1.77E+00	1.1
Cerasus 1	lichen	C1-L6	PA	2017/7/14	6.65E+01 ± 2.95E+00	4.34E+02 ± 6.79E+00	1.14E+01 ± 5.08E-01	7.46E+01 ± 1.17E+00	1.1
Cerasus 1	lichen	C1-L7	PC	2017/7/14	1.25E+02 ± 4.06E+00	9.03E+02 ± 1.06E+01	2.10E+01 ± 6.82E-01	1.52E+02 ± 1.77E+00	1.0
Cerasus 1	lichen	C1-L8	PC	2017/7/14	1.22E+02 ± 3.62E+00	8.69E+02 ± 9.61E+00	2.27E+01 ± 6.73E-01	1.62E+02 ± 1.79E+00	1.0
Cerasus 1	lichen	C1-L9	PA	2017/7/14	2.06E+02 ± 5.21E+00	1.41E+03 ± 1.41E+01	3.37E+01 ± 8.52E-01	2.30E+02 ± 2.31E+00	1.1
Cerasus 2	lichen	C2-L1	PuB	2017/7/14	1.88E+02 ± 4.94E+00	1.34E+03 ± 1.37E+01	3.07E+01 ± 8.07E-01	2.19E+02 ± 2.24E+00	1.0
Cerasus 2	lichen	C2-L2	PuB	2017/7/14	2.59E+02 ± 6.61E+00	1.79E+03 ± 1.81E+01	4.02E+01 ± 1.03E+00	2.78E+02 ± 2.82E+00	1.1
Cerasus 2	lichen	C2-L3	PuB	2017/7/14	3.48E+02 ± 7.11E+00	2.41E+03 ± 2.11E+01	5.44E+01 ± 1.11E+00	3.77E+02 ± 3.30E+00	1.1
Cerasus 2	lichen	C2-L4	PA	2017/7/14	1.55E+02 ± 5.05E+00	1.14E+03 ± 1.35E+01	2.86E+01 ± 9.32E-01	2.11E+02 ± 2.49E+00	0.99
Cerasus 2	lichen	C2-L5	PC	2017/7/14	1.82E+02 ± 5.76E+00	1.28E+03 ± 1.51E+01	2.74E+01 ± 8.66E-01	1.92E+02 ± 2.27E+00	1.0
Cerasus 2	lichen	C2-L6	PC	2017/7/14	1.06E+02 ± 3.49E+00	7.31E+02 ± 8.93E+00	2.03E+01 ± 6.68E-01	1.40E+02 ± 1.71E+00	1.1
Cerasus 2	lichen	C2-L7	DA	2017/7/14	1.54E+02 ± 7.82E+00	1.13E+03 ± 1.90E+01	2.63E+01 ± 1.34E+00	1.93E+02 ± 3.25E+00	0.99
Cerasus 2	lichen	C2-L8	DA	2017/7/14	2.19E+02 ± 5.25E+00	1.55E+03 ± 1.49E+01	4.43E+01 ± 1.06E+00	3.14E+02 ± 3.01E+00	1.0
Cerasus 2	lichen	C2-L9	PuB	2017/7/14	1.24E+02 ± 4.42E+00	9.01E+02 ± 1.14E+01	2.19E+01 ± 7.81E-01	1.59E+02 ± 2.02E+00	1.0
Cerasus 2	lichen	C2-L10	DA	2017/7/14	2.90E+02 ± 8.06E+00	2.09E+03 ± 2.22E+01	4.84E+01 ± 1.35E+00	3.49E+02 ± 3.70E+00	1.0
Cerasus 2	lichen	C2-L11	PC	2017/7/14	7.99E+01 ± 2.73E+00	5.79E+02 ± 7.03E+00	1.78E+01 ± 6.07E-01	1.29E+02 ± 1.56E+00	1.0
Cerasus 3	lichen	C3-L1	FC	2017/7/14	4.06E+01 ± 2.44E+00	3.07E+02 ± 5.82E+00	7.05E+00 ± 4.24E-01	5.33E+01 ± 1.01E+00	0.96
Cerasus 3	lichen	C3-L2	PC	2017/7/14	1.16E+02 ± 4.52E+00	8.52E+02 ± 1.16E+01	2.26E+01 ± 8.79E-01	1.66E+02 ± 2.25E+00	0.99
Cerasus 3	lichen	C3-L3	PC	2017/7/14	7.79E+01 ± 4.24E+00	6.20E+02 ± 1.04E+01	1.17E+01 ± 6.40E-01	9.34E+01 ± 1.56E+00	0.91
Cerasus 3	lichen	C3-L4	PC	2017/7/14	2.14E+02 ± 4.07E+00	1.49E+03 ± 1.25E+01	5.94E+01 ± 1.13E+00	4.14E+02 ± 3.47E+00	1.0
Cerasus 3	lichen	C3-L5	PT	2017/7/14	1.65E+02 ± 3.95E+00	1.13E+03 ± 1.08E+01	3.98E+01 ± 9.53E-01	2.72E+02 ± 2.61E+00	1.1
Cerasus 3	lichen	C3-L6	PT	2017/7/14	7.61E+01 ± 2.72E+00	5.47E+02 ± 6.84E+00	1.91E+01 ± 6.82E-01	1.37E+02 ± 1.71E+00	1.0
Cerasus 1	bark	C1-B1	-	2017/7/14	1.93E+01 ± 9.53E-01	1.36E+02 ± 2.27E+00	9.71E+00 ± 4.79E-01	6.84E+01 ± 1.14E+00	1.0
Cerasus 1	bark	C1-B2	-	2017/7/14	1.20E+01 ± 8.40E-01	8.95E+01 ± 1.94E+00	5.97E+00 ± 4.18E-01	4.45E+01 ± 9.64E-01	0.98
Cerasus 1	bark	C1-B3	-	2017/7/14	6.87E+00 ± 7.89E-01	5.81E+01 ± 1.81E+00	2.82E+00 ± 3.24E-01	2.39E+01 ± 7.44E-01	0.86
Cerasus 1	bark	C1-B4	-	2017/7/14	4.70E+00 ± 5.97E-01	3.78E+01 ± 1.23E+00	2.84E+00 ± 3.61E-01	2.28E+01 ± 7.43E-01	0.91
Cerasus 1	bark	C1-B5	-	2017/7/14	3.19E+00 ± 3.72E-01	2.50E+01 ± 8.18E-01	2.08E+00 ± 2.43E-01	1.63E+01 ± 5.34E-01	0.93
Cerasus 1	bark	C1-B6	-	2017/7/14	5.15E+00 ± 5.04E-01	3.38E+01 ± 9.95E-01	3.69E+00 ± 3.61E-01	2.42E+01 ± 7.13E-01	1.1
Cerasus 1	bark	C1-B7	-	2017/7/14	5.58E+00 ± 4.72E-01	4.62E+01 ± 1.12E+00	4.43E+00 ± 3.75E-01	3.67E+01 ± 8.91E-01	0.88
Cerasus 2	bark	C2-B1	-	2017/7/14	1.63E+01 ± 5.34E-01	1.12E+02 ± 1.34E+00	1.79E+01 ± 5.88E-01	1.23E+02 ± 1.48E+00	1.1
Cerasus 2	bark	C2-B2	-	2017/7/14	5.59E+00 ± 2.47E-01	3.96E+01 ± 5.92E-01	1.10E+01 ± 4.85E-01	7.77E+01 ± 1.16E+00	1.0
Cerasus 2	bark	C2-B3	-	2017/7/14	1.39E+01 ± 4.25E-01	8.77E+01 ± 1.04E+00	2.56E+01 ± 7.83E-01	1.61E+02 ± 1.91E+00	1.2
Cerasus 2	bark	C2-B4	-	2017/7/14	7.20E+00 ± 3.47E-01	5.54E+01 ± 8.60E-01	8.65E+00 ± 4.17E-01	6.65E+01 ± 1.03E+00	0.95
Cerasus 2	bark	C2-B5	-	2017/7/14	4.94E+00 ± 3.32E-01	3.27E+01 ± 7.02E-01	4.89E+00 ± 3.29E-01	3.24E+01 ± 6.95E-01	1.1
Cerasus 2	bark	C2-B6	-	2017/7/14	3.45E+00 ± 2.20E-01	2.63E+01 ± 5.04E-01	6.27E+00 ± 4.00E-01	4.78E+01 ± 9.16E-01	0.95
Cerasus 3	bark	C3-B1	-	2017/7/14	4.82E+00 ± 3.77E-01	3.29E+01 ± 8.10E-01	3.30E+00 ± 2.58E-01	2.25E+01 ± 5.55E-01	1.1
Cerasus 3	bark	C3-B2	-	2017/7/14	4.39E+00 ± 3.17E-01	3.22E+01 ± 6.95E-01	4.61E+00 ± 3.33E-01	3.38E+01 ± 7.29E-01	0.99
Cerasus 3	bark	C3-B3	-	2017/7/14	1.87E+01 ± 6.16E-01	1.25E+02 ± 1.53E+00	1.92E+01 ± 6.32E-01	1.28E+02 ± 1.57E+00	1.1
Cerasus 3	bark	C3-B4	-	2017/7/14	4.84E+00 ± 4.86E-01	3.63E+01 ± 9.77E-01	3.24E+00 ± 3.25E-01	2.43E+01 ± 6.55E-01	0.97

Sample type	Sample ID	Sampling date	Radiocaesium activity concentrations (kBq/kg)		Radiocaesium inventories (kBq/m^{2})		Activity ratio ^{134}Cs/^{137}Cs
			^{134}Cs	^{137}Cs	^{134}Cs	^{137}Cs	
surface soil	S1	2017/7/14	4.15E+01 ± 2.73E-01	3.09E+02 ± 1.80E+00	1.18E+03 ± 7.81E+00	8.83E+03 ± 5.13E+01	0.98
surface soil	S2	2017/7/14	3.39E+01 ± 2.22E-01	2.55E+02 ± 1.48E+00	1.47E+03 ± 9.65E+00	1.11E+04 ± 6.44E+01	0.97
surface soil	S3	2017/7/14	2.40E+01 ± 1.69E-01	1.79E+02 ± 1.05E+00	7.80E+02 ± 5.48E+00	5.83E+03 ± 3.42E+01	0.97
surface soil	S4	2017/7/14	1.43E+01 ± 1.08E-01	1.08E+02 ± 6.45E-01	6.40E+02 ± 4.81E+00	4.85E+03 ± 2.88E+01	0.96
surface soil	S5	2017/7/14	1.34E+01 ± 1.02E-01	1.01E+02 ± 6.02E-01	6.21E+02 ± 4.70E+00	4.67E+03 ± 2.78E+01	0.97
surface soil	S6	2017/7/14	1.13E+01 ± 8.97E-02	8.61E+01 ± 5.17E-01	4.85E+02 ± 3.84E+00	3.69E+03 ± 2.21E+01	0.96
surface soil	S7	2017/7/14	1.19E+01 ± 9.38E-02	8.94E+01 ± 5.37E-01	4.92E+02 ± 3.88E+00	3.70E+03 ± 2.22E+01	0.97
surface soil	S8	2017/7/14	3.28E+00 ± 3.98E-02	2.53E+01 ± 1.72E-01	1.46E+02 ± 1.77E+00	1.13E+03 ± 7.67E+00	0.95
surface soil	S9	2017/7/14	1.23E+01 ± 9.87E-02	9.31E+01 ± 5.61E-01	5.21E+02 ± 4.17E+00	3.93E+03 ± 2.37E+01	0.97
surface soil	S10	2017/7/14	2.19E+01 ± 1.55E-01	1.65E+02 ± 9.71E-01	7.85E+02 ± 5.54E+00	5.92E+03 ± 3.48E+01	0.97
surface soil	S11	2017/7/14	4.74E+01 ± 3.03E-01	3.58E+02 ± 2.07E+00	1.72E+03 ± 1.10E+01	1.30E+04 ± 7.50E+01	0.96
surface soil	S12	2017/7/14	2.01E+00 ± 2.84E-02	1.56E+01 ± 1.13E-01	9.93E+01 ± 1.41E+00	7.70E+02 ± 5.59E+00	0.94
surface soil	S13	2017/7/14	1.74E+01 ± 1.28E-01	1.31E+02 ± 7.76E-01	7.61E+02 ± 5.63E+00	5.74E+03 ± 3.40E+01	0.97
surface soil	S14	2017/7/14	1.96E+01 ± 1.42E-01	1.47E+02 ± 8.67E-01	7.02E+02 ± 5.09E+00	5.27E+03 ± 3.11E+01	0.97
surface soil	S15	2017/7/14	1.60E+01 ± 1.19E-01	1.21E+02 ± 7.19E-01	7.05E+02 ± 5.24E+00	5.34E+03 ± 3.17E+01	0.96
surface soil	S16	2017/7/14	3.57E+01 ± 2.37E-01	2.72E+02 ± 1.58E+00	1.19E+03 ± 7.90E+00	9.08E+03 ± 5.28E+01	0.95
surface soil	S17	2017/7/14	2.82E+01 ± 1.89E-01	2.14E+02 ± 1.25E+00	1.31E+03 ± 8.79E+00	9.95E+03 ± 5.80E+01	0.96
surface soil	S18	2017/7/14	3.09E+01 ± 2.07E-01	2.34E+02 ± 1.36E+00	1.31E+03 ± 8.75E+00	9.90E+03 ± 5.76E+01	0.96
surface soil	S19	2017/7/14	2.39E+01 ± 1.69E-01	1.79E+02 ± 1.05E+00	7.91E+02 ± 5.59E+00	5.94E+03 ± 3.49E+01	0.97
surface soil	S20	2017/7/14	1.29E+01 ± 9.92E-02	9.83E+01 ± 5.87E-01	5.46E+02 ± 4.21E+00	4.17E+03 ± 2.49E+01	0.95
surface soil	S21	2017/7/14	2.36E+01 ± 1.64E-01	1.79E+02 ± 1.05E+00	8.79E+02 ± 6.10E+00	6.66E+03 ± 3.90E+01	0.96
surface soil	S22	2017/7/14	1.65E+01 ± 1.22E-01	1.25E+02 ± 7.37E-01	6.70E+02 ± 4.95E+00	5.06E+03 ± 3.00E+01	0.96
surface soil	S23	2017/7/14	1.37E+01 ± 1.07E-01	1.03E+02 ± 6.18E-01	4.98E+02 ± 3.88E+00	3.74E+03 ± 2.24E+01	0.97
surface soil	S24	2017/7/14	6.47E+00 ± 5.98E-02	4.93E+01 ± 3.07E-01	2.84E+02 ± 2.63E+00	2.17E+03 ± 1.35E+01	0.96
surface soil	S25	2017/7/14	2.29E+01 ± 1.57E-01	1.70E+02 ± 9.94E-01	1.00E+03 ± 6.87E+00	7.42E+03 ± 4.34E+01	0.98
organic layer	Lit1	2017/7/14	2.83E+01 ± 3.57E-01	2.11E+02 ± 1.46E+00	4.02E+00 ± 5.07E-02	2.99E+01 ± 2.07E-01	0.98
organic layer	Lit2	2017/7/14	2.15E+01 ± 3.11E-01	1.59E+02 ± 1.17E+00	2.83E+00 ± 4.09E-02	2.09E+01 ± 1.53E-01	0.98
organic layer	Lit3	2017/7/14	2.90E+01 ± 2.86E-01	2.11E+02 ± 1.34E+00	8.45E+00 ± 8.33E-02	6.14E+01 ± 3.91E-01	1.0
organic layer	Lit4	2017/7/14	2.55E+01 ± 2.58E-01	1.86E+02 ± 1.19E+00	7.94E+00 ± 8.03E-02	5.80E+01 ± 3.72E-01	1.0
organic layer	Lit5	2017/7/14	2.10E+01 ± 2.28E-01	1.55E+02 ± 1.01E+00	6.88E+00 ± 7.46E-02	5.06E+01 ± 3.32E-01	0.99
organic layer	Lit6	2017/7/14	4.35E+01 ± 3.54E-01	3.20E+02 ± 1.94E+00	1.75E+01 ± 1.42E-01	1.29E+02 ± 7.81E-01	0.99
organic layer	Lit7	2017/7/14	2.34E+01 ± 2.41E-01	1.72E+02 ± 1.11E+00	8.60E+00 ± 8.84E-02	6.32E+01 ± 4.08E-01	0.99
organic layer	Lit8	2017/7/14	2.20E+01 ± 5.08E-01	1.53E+02 ± 1.49E+00	8.79E-01 ± 2.03E-02	6.14E+00 ± 5.95E-02	1.0
organic layer	Lit9	2017/7/14	4.80E+01 ± 4.40E-01	3.51E+02 ± 2.18E+00	9.04E+00 ± 8.29E-02	6.62E+01 ± 4.11E-01	0.99
organic layer	Lit10	2017/7/14	2.69E+01 ± 3.30E-01	1.95E+02 ± 1.33E+00	4.06E+00 ± 4.99E-02	2.94E+01 ± 2.01E-01	1.0
organic layer	Lit11	2017/7/14	3.48E+01 ± 2.96E-01	2.57E+02 ± 1.57E+00	1.58E+01 ± 1.34E-01	1.16E+02 ± 7.12E-01	0.99
organic layer	Lit12	2017/7/14	1.84E+01 ± 2.12E-01	1.34E+02 ± 8.97E-01	5.27E+00 ± 6.08E-02	3.84E+01 ± 2.57E-01	1.0
organic layer	Lit13	2017/7/14	1.88E+01 ± 2.25E-01	1.37E+02 ± 9.32E-01	4.93E+00 ± 5.89E-02	3.60E+01 ± 2.44E-01	1.0
organic layer	Lit14	2017/7/14	5.21E+01 ± 4.25E-01	3.87E+02 ± 2.34E+00	1.60E+01 ± 1.31E-01	1.19E+02 ± 7.19E-01	0.98
organic layer	Lit15	2017/7/14	4.70E+01 ± 3.73E-01	3.41E+02 ± 2.05E+00	1.65E+01 ± 1.31E-01	1.20E+02 ± 7.19E-01	1.0
organic layer	Lit16	2017/7/14	2.12E+01 ± 2.80E-01	1.53E+02 ± 1.08E+00	3.46E+00 ± 4.57E-02	2.49E+01 ± 1.76E-01	1.0
organic layer	Lit17	2017/7/14	3.11E+01 ± 3.56E-01	2.24E+02 ± 1.50E+00	5.06E+00 ± 5.79E-02	3.64E+01 ± 2.43E-01	1.0
organic layer	Lit18	2017/7/14	5.92E+01 ± 4.28E-01	4.37E+02 ± 2.58E+00	3.23E+01 ± 2.34E-01	2.38E+02 ± 1.41E+00	0.99
organic layer	Lit19	2017/7/14	2.55E+01 ± 2.42E-01	1.90E+02 ± 1.20E+00	1.13E+01 ± 1.07E-01	8.40E+01 ± 5.28E-01	0.98
organic layer	Lit20	2017/7/14	9.79E+01 ± 6.88E-01	7.23E+02 ± 4.25E+00	3.44E+01 ± 2.42E-01	2.54E+02 ± 1.49E+00	0.98
organic layer	Lit21	2017/7/14	3.54E+01 ± 2.86E-01	2.61E+02 ± 1.57E+00	1.78E+01 ± 1.44E-01	1.31E+02 ± 7.91E-01	0.99
organic layer	Lit22	2017/7/14	1.82E+01 ± 2.69E-01	1.32E+02 ± 9.82E-01	2.62E+00 ± 3.86E-02	1.90E+01 ± 1.41E-01	1.0
organic layer	Lit23	2017/7/14	1.71E+01 ± 2.40E-01	1.26E+02 ± 9.11E-01	3.18E+00 ± 4.45E-02	2.34E+01 ± 1.69E-01	0.99
