# Supplementary material for: Radiocaesium accumulation capacity of epiphytic lichens and adjacent barks collected at the perimeter boundary site of the Fukushima Dai-ichi Nuclear Power Station
Source: PLoS One. 2021 May 24;16(5):e0251828. doi: 10.1371/journal.pone.0251828 (PMC8143426; doi:10.1371/journal.pone.0251828)
Supplement: S2 Table — (PDF) [file pone.0251828.s002.pdf]

S2 Table. GM counting values and inventories of lichen and bark samples.

Substrate (tree)	Sample type	Sample ID	Species (for lichens)	Sampling date	GM counting value (kcpm)	Activity per unit area (kBq m^{-2})
Zelkova 1	lichen	Z1-L1	PuB	2017/6/22	16.5	4.3E+02
Zelkova 1	lichen	Z1-L2	PC	2017/6/22	16.2	4.1E+02
Zelkova 1	lichen	Z1-L3	PuB	2017/6/22	14.2	3.4E+02
Zelkova 1	lichen	Z1-L4	PuB	2017/6/22	11.2	2.4E+02
Zelkova 1	lichen	Z1-L5	PC	2017/6/22	16.6	4.3E+02
Zelkova 1	lichen	Z1-L6	PuB	2017/6/22	15.2	3.8E+02
Zelkova 1	lichen	Z1-L7	DA	2017/6/22	14.8	3.6E+02
Zelkova 1	lichen	Z1-L8	PuB	2017/6/22	19.2	5.2E+02
Zelkova 1	lichen	Z1-L9	PuB	2017/6/22	13.0	3.0E+02
Zelkova 1	lichen	Z1-L10	PuB	2017/6/22	17.6	4.7E+02
Zelkova 1	lichen	Z1-L11	PC	2017/6/22	10.9	2.3E+02
Zelkova 1	lichen	Z1-L12	DA	2017/6/22	18.2	4.9E+02
Zelkova 1	lichen	Z1-L13	PC	2017/6/22	14.7	3.6E+02
Zelkova 1	lichen	Z1-L14	PC	2017/6/22	11.1	2.3E+02
Zelkova 1	lichen	Z1-L15	PuB	2017/6/22	17.2	4.5E+02
Zelkova 1	lichen	Z1-L16	PC	2017/6/22	16.1	4.1E+02
Zelkova 1	lichen	Z1-L17	PuB	2017/6/22	12.0	2.6E+02
Zelkova 2	lichen	Z2-L1	PC	2017/6/23	16.2	4.0E+02
Zelkova 2	lichen	Z2-L2	PC	2017/6/23	15.5	3.7E+02
Zelkova 2	lichen	Z2-L3	PA	2017/6/23	12.5	2.7E+02
Zelkova 2	lichen	Z2-L4	PC	2017/6/23	20.8	5.6E+02
Zelkova 2	lichen	Z2-L5	PC	2017/6/23	12.4	2.6E+02
Zelkova 2	lichen	Z2-L6	PM	2017/6/23	13.2	2.9E+02
Zelkova 2	lichen	Z2-L7	PuB	2017/6/23	11.1	2.2E+02
Zelkova 2	lichen	Z2-L8	PuB	2017/6/23	10.2	1.8E+02
Zelkova 2	lichen	Z2-L9	PC	2017/6/23	20.1	5.4E+02
Zelkova 2	lichen	Z2-L10	PuB	2017/6/23	15.3	3.7E+02
Zelkova 2	lichen	Z2-L11	PuB	2017/6/23	20.4	5.5E+02
Zelkova 2	lichen	Z2-L12	PuB	2017/6/23	15.2	3.6E+02
Zelkova 2	lichen	Z2-L13	CA	2017/6/23	10.3	1.9E+02
Zelkova 2	lichen	Z2-L14	PuB	2017/6/23	10.1	1.8E+02
Zelkova 3	lichen	Z3-L1	PuB	2017/6/23	16.2	3.6E+02
Zelkova 3	lichen	Z3-L2	PuB	2017/6/23	16.5	3.8E+02
Zelkova 3	lichen	Z3-L3	PC	2017/6/23	16.2	3.6E+02
Zelkova 3	lichen	Z3-L4	PuB	2017/6/23	13.8	2.8E+02
Zelkova 3	lichen	Z3-L5	ML	2017/6/23	27.5	7.7E+02
Zelkova 3	lichen	Z3-L6	PuB	2017/6/23	18.7	4.5E+02
Zelkova 3	lichen	Z3-L7	DA	2017/6/23	19.2	4.7E+02
Zelkova 3	lichen	Z3-L8	ML	2017/6/23	MD	MD
Zelkova 1	bark	Z1-B1	-	2017/6/22	6.3	6.1E+01
Zelkova 1	bark	Z1-B2	-	2017/6/22	8.4	1.4E+02
Zelkova 1	bark	Z1-B3	-	2017/6/22	4.5	LOQ
Zelkova 1	bark	Z1-B4	-	2017/6/22	4.1	LOQ
Zelkova 1	bark	Z1-B5	-	2017/6/22	7.5	1.0E+02
Zelkova 1	bark	Z1-B6	-	2017/6/22	6.7	7.6E+01
Zelkova 1	bark	Z1-B7	-	2017/6/22	5.8	4.4E+01
Zelkova 1	bark	Z1-B8	-	2017/6/22	8.3	1.3E+02
Zelkova 1	bark	Z1-B9	-	2017/6/22	7.0	8.5E+01
Zelkova 1	bark	Z1-B10	-	2017/6/22	8.0	1.2E+02
Zelkova 1	bark	Z1-B11	-	2017/6/22	6.1	5.3E+01
Zelkova 2	bark	Z2-B1	-	2017/6/23	5.6	1.9E+01
Zelkova 2	bark	Z2-B2	-	2017/6/23	6.1	3.7E+01
Zelkova 2	bark	Z2-B3	-	2017/6/23	6.7	5.8E+01
Zelkova 2	bark	Z2-B4	-	2017/6/23	5.3	8.2E+00
Zelkova 2	bark	Z2-B5	-	2017/6/23	4.7	LOQ
Zelkova 2	bark	Z2-B6	-	2017/6/23	7.1	7.3E+01
Zelkova 2	bark	Z2-B7	-	2017/6/23	8.1	1.1E+02
Zelkova 2	bark	Z2-B8	-	2017/6/23	7.1	7.3E+01
Zelkova 2	bark	Z2-B9	-	2017/6/23	8.8	1.3E+02
Zelkova 2	bark	Z2-B10	-	2017/6/23	4.5	LOQ
Zelkova 2	bark	Z2-B11	-	2017/6/23	4.7	LOQ
Zelkova 3	bark	Z3-B1	-	2017/6/23	8.8	1.0E+02
Zelkova 3	bark	Z3-B2	-	2017/6/23	7.7	6.0E+01
Zelkova 3	bark	Z3-B3	-	2017/6/23	16.1	3.6E+02
Zelkova 3	bark	Z3-B4	-	2017/6/23	14.9	3.2E+02
Zelkova 3	bark	Z3-B5	-	2017/6/23	MD	MD
Cerasus 1	lichen	C1-L1	PA	2017/7/14	13.8	3.1E+02
Cerasus 1	lichen	C1-L2	PA	2017/7/14	10.1	1.8E+02
Cerasus 1	lichen	C1-L3	PM	2017/7/14	11.3	2.2E+02
Cerasus 1	lichen	C1-L4	PT	2017/7/14	7.5	8.3E+01
Cerasus 1	lichen	C1-L5	PA	2017/7/14	10.3	1.8E+02
Cerasus 1	lichen	C1-L6	PA	2017/7/14	7.5	8.3E+01
Cerasus 1	lichen	C1-L7	PC	2017/7/14	9.7	1.6E+02
Cerasus 1	lichen	C1-L8	PC	2017/7/14	9.5	1.5E+02
Cerasus 1	lichen	C1-L9	PA	2017/7/14	9.5	1.5E+02
Cerasus 2	lichen	C2-L1	PuB	2017/7/14	13.1	2.9E+02
Cerasus 2	lichen	C2-L2	PuB	2017/7/14	12.6	2.8E+02
Cerasus 2	lichen	C2-L3	PuB	2017/7/14	14.5	3.4E+02
Cerasus 2	lichen	C2-L4	PA	2017/7/14	11.2	2.3E+02
Cerasus 2	lichen	C2-L5	PC	2017/7/14	10.9	2.2E+02
Cerasus 2	lichen	C2-L6	PC	2017/7/14	8.3	1.2E+02
Cerasus 2	lichen	C2-L7	DA	2017/7/14	8.0	1.1E+02
Cerasus 2	lichen	C2-L8	DA	2017/7/14	10.5	2.0E+02
Cerasus 2	lichen	C2-L9	PuB	2017/7/14	13.4	3.1E+02
Cerasus 2	lichen	C2-L10	DA	2017/7/14	12.9	2.9E+02
Cerasus 2	lichen	C2-L11	PC	2017/7/14	8.9	1.4E+02
Cerasus 3	lichen	C3-L1	FC	2017/7/14	7.1	1.0E+02
Cerasus 3	lichen	C3-L2	PC	2017/7/14	7.9	1.3E+02
Cerasus 3	lichen	C3-L3	PC	2017/7/14	7.3	1.1E+02
Cerasus 3	lichen	C3-L4	PC	2017/7/14	17.2	4.6E+02
Cerasus 3	lichen	C3-L5	PT	2017/7/14	11.2	2.5E+02
Cerasus 3	lichen	C3-L6	PT	2017/7/14	10.5	2.2E+02
Cerasus 1	bark	C1-B1	-	2017/7/14	6.3	4.0E+01
Cerasus 1	bark	C1-B2	-	2017/7/14	5.1	LOQ
Cerasus 1	bark	C1-B3	-	2017/7/14	5.6	1.5E+01
Cerasus 1	bark	C1-B4	-	2017/7/14	4.6	LOQ
Cerasus 1	bark	C1-B5	-	2017/7/14	4.2	LOQ
Cerasus 1	bark	C1-B6	-	2017/7/14	5.2	7.2E-01
Cerasus 1	bark	C1-B7	-	2017/7/14	5.2	7.2E-01
Cerasus 2	bark	C2-B1	-	2017/7/14	7.8	1.0E+02
Cerasus 2	bark	C2-B2	-	2017/7/14	6.4	5.4E+01
Cerasus 2	bark	C2-B3	-	2017/7/14	7.0	7.6E+01
Cerasus 2	bark	C2-B4	-	2017/7/14	7.0	7.6E+01
Cerasus 2	bark	C2-B5	-	2017/7/14	5.6	2.6E+01
Cerasus 2	bark	C2-B6	-	2017/7/14	5.9	3.7E+01
Cerasus 3	bark	C3-B1	-	2017/7/14	5.2	3.4E+01
Cerasus 3	bark	C3-B2	-	2017/7/14	5.0	2.6E+01
Cerasus 3	bark	C3-B3	-	2017/7/14	5.0	2.6E+01
Cerasus 3	bark	C3-B4	-	2017/7/14	5.0	2.6E+01

MD = Missing value

LOQ = Limit of quantification
